# Quantifying the Impact of Human Leukocyte Antigen on the Human Gut Microbiota

**DOI:** 10.1128/mSphere.00476-21

**Published:** 2021-08-11

**Authors:** Stijn P. Andeweg, Can Keşmir, Bas E. Dutilh

**Affiliations:** a Theoretical Biology and Bioinformatics, Science for Life, Utrecht University, Utrecht, the Netherlands; University of Utah

**Keywords:** gut microbiome, human leukocyte antigen, immune-microbiome interactions, quantitative biology

## Abstract

The composition of the gut microbiota is affected by a number of factors, including the innate and adaptive immune system. The major histocompatibility complex (MHC), or the human leukocyte antigen (HLA) in humans, performs an essential role in vertebrate immunity and is very polymorphic in different populations. HLA determines the specificity of T lymphocyte and natural killer (NK) cell responses, including those against the commensal bacteria present in the human gut. Thus, it is likely that our HLA molecules, and thereby the adaptive immune response, can shape the composition of our microbiota. Here, we investigated the effect of HLA haplotype on the microbiota composition. We performed HLA typing and microbiota composition analyses on 3,002 public human gut microbiome data sets. We found that individuals with functionally similar HLA molecules are also similar in their microbiota composition. Our results show a statistical association between host HLA haplotype and gut microbiota composition. Because the HLA haplotype is a readily measurable parameter of the human immune system, these results open the door to incorporating the genetics of the immune system into predictive microbiome models.

**IMPORTANCE** The microorganisms that live in the digestive tracts of humans, known as the gut microbiota, are essential for hosts’ survival, as they support crucial functions. For example, they support the host in facilitating the uptake of nutrients and give colonization resistance against pathogens. The composition of the gut microbiota varies among humans. Studies have proposed multiple factors driving the observed variation, including diet, lifestyle, and health condition. Another major influence on the microbiota is the host’s genetic background. We hypothesized the immune system to be one of the most important genetic factors driving the differences observed between gut microbiotas. Therefore, we searched for a link between the polymorphic molecules that shape human immune responses and the composition of the microbiota. HLA molecules are the most polymorphic molecules in our genome and therefore makes an excellent candidate to test such an association. To our knowledge for the first time, our results indicate a significant impact of the HLA on the human gut microbiota.

## INTRODUCTION

The gut microbiota is essential for the existence of the mammalian host and performs crucial functions, such as nutrition and colonization resistance against pathogens (1, 2). In addition, commensal bacteria shape host immunity (3, 4): commensal bacteria influence the development and homeostasis of the immune system, and in the absence of a gut microbiota the innate and adaptive immune system is impaired (5). These functions are fulfilled by the 10^13^ to 10^14^ bacteria making up the human gut microbiota of a typical adult (6). The composition of the gut microbiota varies substantially over the human population (7, 8), and numerous exogenous and intrinsic factors are proposed to influence the microbial community composition, leading to observed microbiome diversity (9, 10). These factors include dietary components, mode of delivery during birth, breastfeeding, disease history, and host genetics (10–15). However, the greater part of population diversity is still unexplained. To obtain a better understanding of the microbiota composition and function, a comprehensive list of associated factors is required.

Studies with monozygotic twins have highlighted the importance of host genetics in shaping the microbiota composition (16, 17), where the immune system is thought to be one of the most important contributing factors (18). The immune system is a well-known but not fully explored mechanism influencing the microbiome (18–21). Conversely, the microbiota also imprints its signature on the host immune system, e.g., by influencing the abundances of mucosa-associated invariant T cells by exposure to microbial antigens (22). Immune response related factors that generate variation between microbiotas are likely to originate from functional differences among polymorphic immune molecules.

Major histocompatibility complex (MHC) genes, known as the human leukocyte antigen (HLA) genes in humans, are one of the most polymorphic genes found in vertebrates (23, 24). They encode cell surface glycoproteins that present antigens to T cells (25–27) and form the first essential step in the generation of an adaptive immune response. Influence of the MHC haplotype on the microbiome has been observed in mice (18, 28–30) and in stickleback (31). In humans, specific polymorphisms in the HLA region have been associated with several common infections (32), and in a genome-wide association study of over 1,500 individuals, two variants located in the HLA region showed suggestive association with microbiome composition or function (12). Moreover, an association with microbiota composition has been observed for the HLA-DQ gene in relation to celiac disease (33, 34). However, other genome-wide association studies did not find associations between HLA and the microbiome among many associated genes (35, 36).

Here, we analyzed 3,002 human gut microbiome data sets and found that HLA haplotype similarity correlates with microbiota similarity. Importantly, functionally stratifying individuals on the basis of HLA-presented microbial antigens improved the association with microbiota similarity. Overall, these results provide the first statistical support for the functional association between HLA and the gut microbiota in humans.

## RESULTS

### Mining gut microbiome data sets.

To link the HLA haplotype to microbiota composition of individuals, we simultaneously determined the presence of human HLA genes and bacterial small-subunit (SSU) rRNA genes in human intestinal samples. We composed a combined reference database consisting of HLA gene sequences and SSU rRNA sequences and used this to map 3,002 gut microbiome data sets extracted from the Sequence Read Archive (SRA). As expected, we observed a large variability in the number of HLA reads recovered from the different data sets (Table 1; also, see Fig. S1A in the supplemental material). This variability could be due to differences in sequencing techniques between studies or due to differences between individuals. We designed a decision tree (see Materials and Methods) to predict the HLA haplotype for the classical HLA class I (HLA-A, HLA-B, and HLA-C) and HLA class II (HLA-DP, HLA-DQ and HLA-DR) genes. The decision tree predicted alleles from replicate samples of the same individual with a mean overall consistency of 75%, and zygosity was predicted with a mean consistency of 80% (Table 1). This performance is particularly notable for the specific alleles, as they were predicted based on a reference database consisting of between 5 (HLA-DQ) and 35 (HLA-B) two-digit alleles depending on the HLA gene, while zygosity was only a binary call (heterozygote/homozygote, i.e., a 50% guessing rate). Together, these results provide a rough reliability estimate for our HLA typing pipeline.

**TABLE 1:**
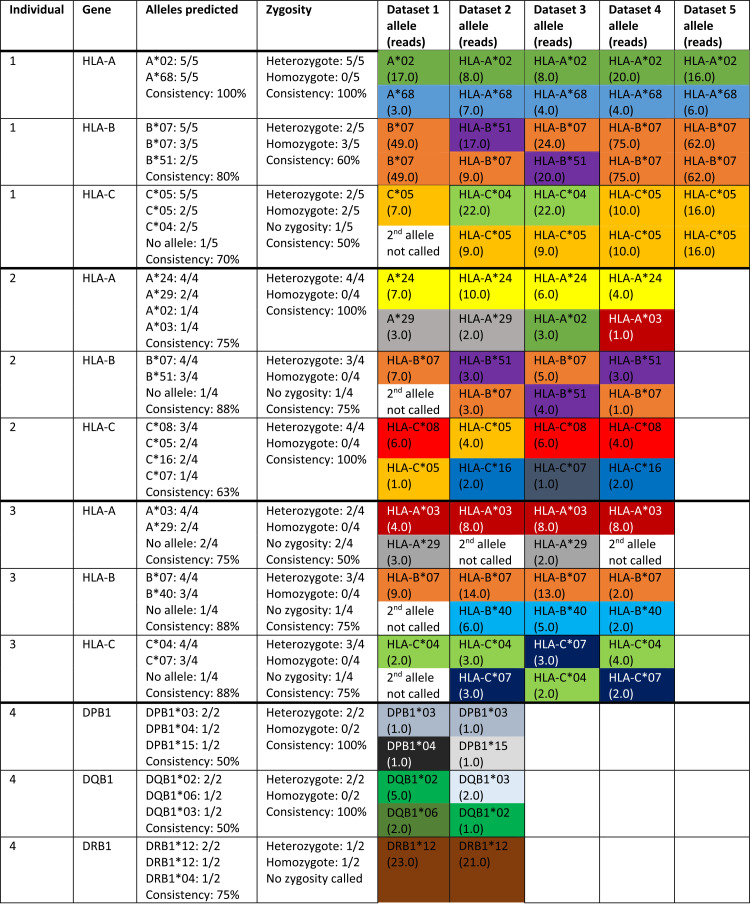
Consistency of HLA prediction over the available data sets from the same individual^*a*^

a HLA predictions from different data sets compared to the most likely HLA alleles prediction of the individual. Each two-digit allele was given a unique color, showing that most alleles were consistently predicted for different individuals. Frequently occurring alleles (e.g., B*07) in this selection of predictions were also observed more frequently in the general population (Fig. S2). In the set of samples from the same individual, no class II predictions were made because of a lack of class II reads found in this data set.

10.1128/mSphere.00476-21.1 FIG S1 Reads mapping to HLA genes in all samples and per group, selected by having a complete profile for the HLA class I or II genes. (A) The number of samples containing different numbers of HLA reads. (B, left) Discontinuous bar plot showing the number of reads mapping to HLA-A, -B, or -C genes for the data sets in the HLA class I group. (B, right) Reads mapping to HLA-DPB1, -DRB1, or -DQB1 genes for the datasets in the HLA class II group. Download FIG S1, TIF file, 0.5 MB. Copyright © 2021 Andeweg et al. 2021 Andeweg et al. https://creativecommons.org/licenses/by/4.0/ This content is distributed under the terms of the Creative Commons Attribution 4.0 International license.

10.1128/mSphere.00476-21.2 FIG S2 HLA frequencies compared between current study and National Marrow Donor Program (NMDP). (A to C) The HLA frequency distribution of the 127 samples selected on HLA class I compared to the HLA frequency of European origin in the NMDP database (41), for HLA-A (A), HLA-B (B), and HLA-C (C). Histogram of the distribution of the HLA class II DQB1 (D) and DRB1 (E) genes compared to the NMDP. The heterozygote/homozygote distribution for the HLA class I and II genes (F). Again, the data are compared to the NMDP zygosity frequencies. Data for the DPB1 gene were absent from the NMDP database. Download FIG S2, TIF file, 1.1 MB. Copyright © 2021 Andeweg et al. 2021 Andeweg et al. https://creativecommons.org/licenses/by/4.0/ This content is distributed under the terms of the Creative Commons Attribution 4.0 International license.

Application of this scheme to all 3,002 data sets resulted in prediction of the complete HLA class I and II profiles for 127 and 309 individuals, respectively (Fig. S1B). HLA class I is expressed in all nucleated cells, while HLA class II expression is restricted to antigen-presenting cells (APCs) (25). Therefore, we expected a higher number of reads mapping to HLA class I in human gut data sets, but the difference between HLA class I and II reads is not significant (*P* = 0.17, two-sample Kolmogorov-Smirnov [KS] test). The observation of similar read counts between HLA class I and II may be explained by a large number of lymphocytes (especially B cells, which have high MHC class II expression) being present in the gut as a part of mucosal immunity (37). Moreover, intestinal epithelial cells also express MHC class II (38), and HLA class I and class II expression levels vary across different cell types (39, 40). Further differences in the gene expression pattern of gut-associated cell types, as well as stochastic differences in, e.g., sampling and analysis protocols, might contribute to the observed variation in HLA mapped reads between the SRA data sets.

### Inferred HLA prevalence reflects the Caucasian population profile.

As an additional quality assessment of our HLA typing pipeline, the predicted population allele frequencies of the HLA class I and class II groups were compared to the HLA prevalence in the National Marrow Donor Program (NMDP) database, a database containing high-resolution HLA allele frequencies (41). The frequency distribution of HLA class I in our data set is similar to that in the NMDP database of Caucasian ethnicity (Pearson’s *r* = 0.806, 0.726, and 0.812 for HLA-A, HLA-B, and HLA-C, respectively), but the frequency distribution of HLA class II is less so (Pearson’s *r* = 0.537 and 0.127 for HLA-DQB1 and HLA-DRB1, respectively) (Fig. S2A to E). Finally, we observed a high frequency of HLA heterozygosity and low frequency of homozygosity for any of the HLA genes (Fig. S2F), consistent with the high polymorphism of HLA genes in the population (41). All genes except HLA-C had zygosity frequencies that were consistent with the NMDP data (Fig. S2F).

### Individuals with similar HLA genes have similar microbiota.

**FIG 1:**
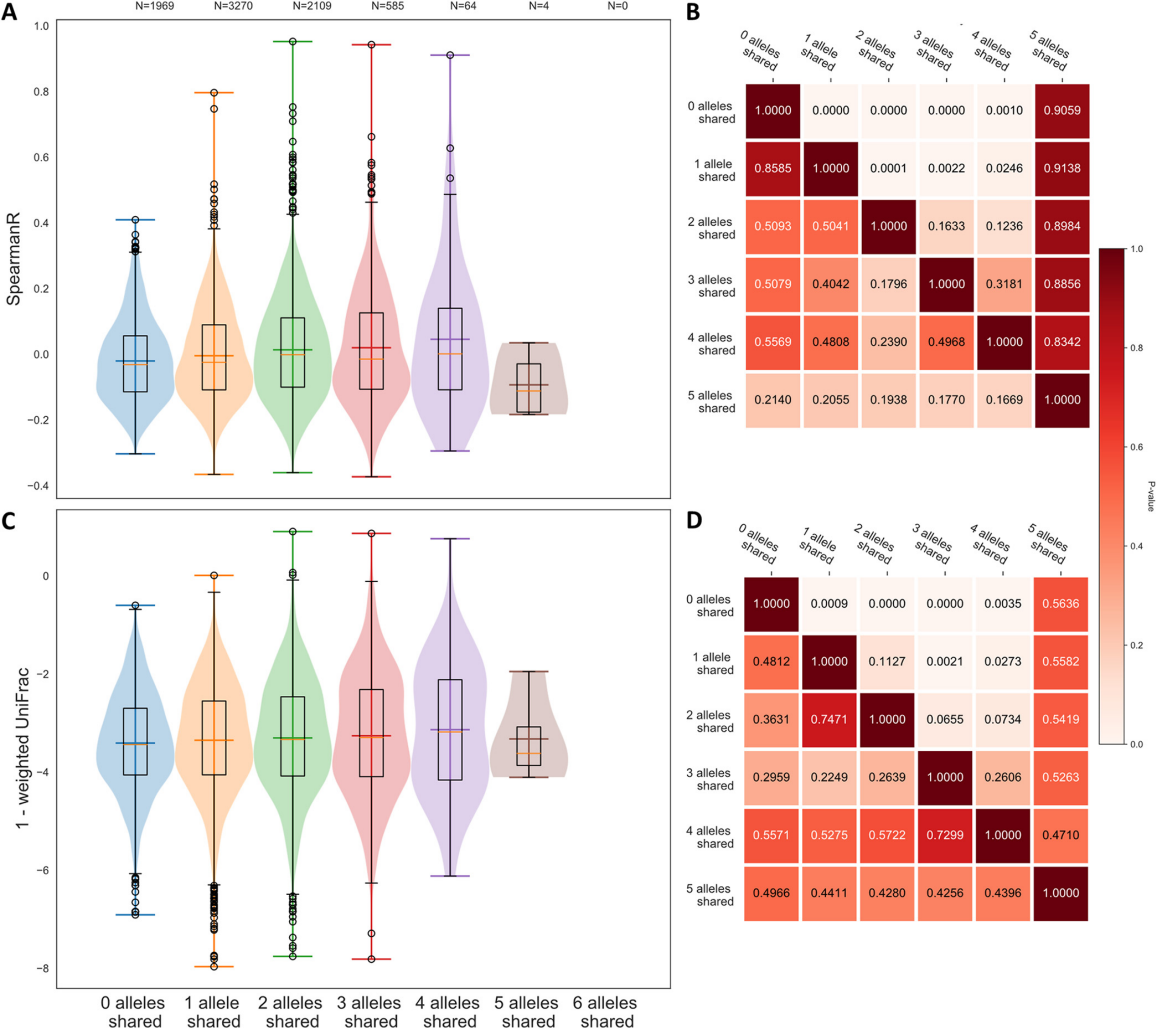
Microbiota beta diversity for sample pairs with an increasing number of shared HLA class I alleles. (A) Spearman rank correlation and (C) weighted UniFrac of the microbiota composition for sample pairs with 0 (blue), 1 (orange), 2 (green), 3 (red), 4 (purple), or 5 (brown) alleles shared. (B and D) Heat maps displaying the *P* values for the two-sample Kolmogorov-Smirnov test under the null hypothesis that the item in the row is drawn from an equal or smaller distribution compared to the item in the column.

Next, we compared microbiota similarity between the individuals in search for a link to similarity in HLA alleles. To do this, we determined the similarity in microbiota composition for pairs of data sets that were stratified by the number of shared HLA class I alleles (Fig. 1). As expected, microbiota similarity was generally higher between individuals who shared more HLA alleles, although the effect size was modest, possibly as a result of the low number of sample pairs with highly similar HLA profiles.

We expect that two individuals with the same HLA molecules may have similar immune control of their microbiota. However, the opposite is not true, i.e., individuals with different sets of HLA molecules can nevertheless generate similar immune responses, because different HLA molecules may have similar binding motifs. To address this issue of functional similarity, HLA molecules have been grouped into supertypes, consisting of HLA molecules that present similar peptides on the cell surface (42). In a first attempt to represent the functional HLA similarity between individuals and increase statistical power at this highly polymorphic gene, we performed association analyses at the level of HLA supertypes. While this increased the number of sample pairs with similar HLA profiles, the relation between HLA similarity and microbiota beta diversity was lost (Fig. S3). This may be because the HLA supertypes are a very generalized representation of HLA molecules, while the functional effects of the HLA molecules on the microbiota composition are likely very specific (43).

10.1128/mSphere.00476-21.3 FIG S3 Microbiota beta diversity for samples with shared HLA class I supertypes. (A) Spearman rank correlation and (C) weighted UniFrac distance for sample pairs with 0 (blue), 1 (orange), 2 (green), 3 (red), or 4 (purple) HLA-A and -B supertypes shared. Heat maps on the right display the *P* values for the two-sample Kolmogorov-Smirnov test under the null hypothesis; the item on the *y* axis is drawn from an equal or smaller distribution than the item on the *x* axis, for Spearman rank (B) and weighted UniFrac (D). Download FIG S3, TIF file, 1.2 MB. Copyright © 2021 Andeweg et al. 2021 Andeweg et al. https://creativecommons.org/licenses/by/4.0/ This content is distributed under the terms of the Creative Commons Attribution 4.0 International license.

**FIG 2:**
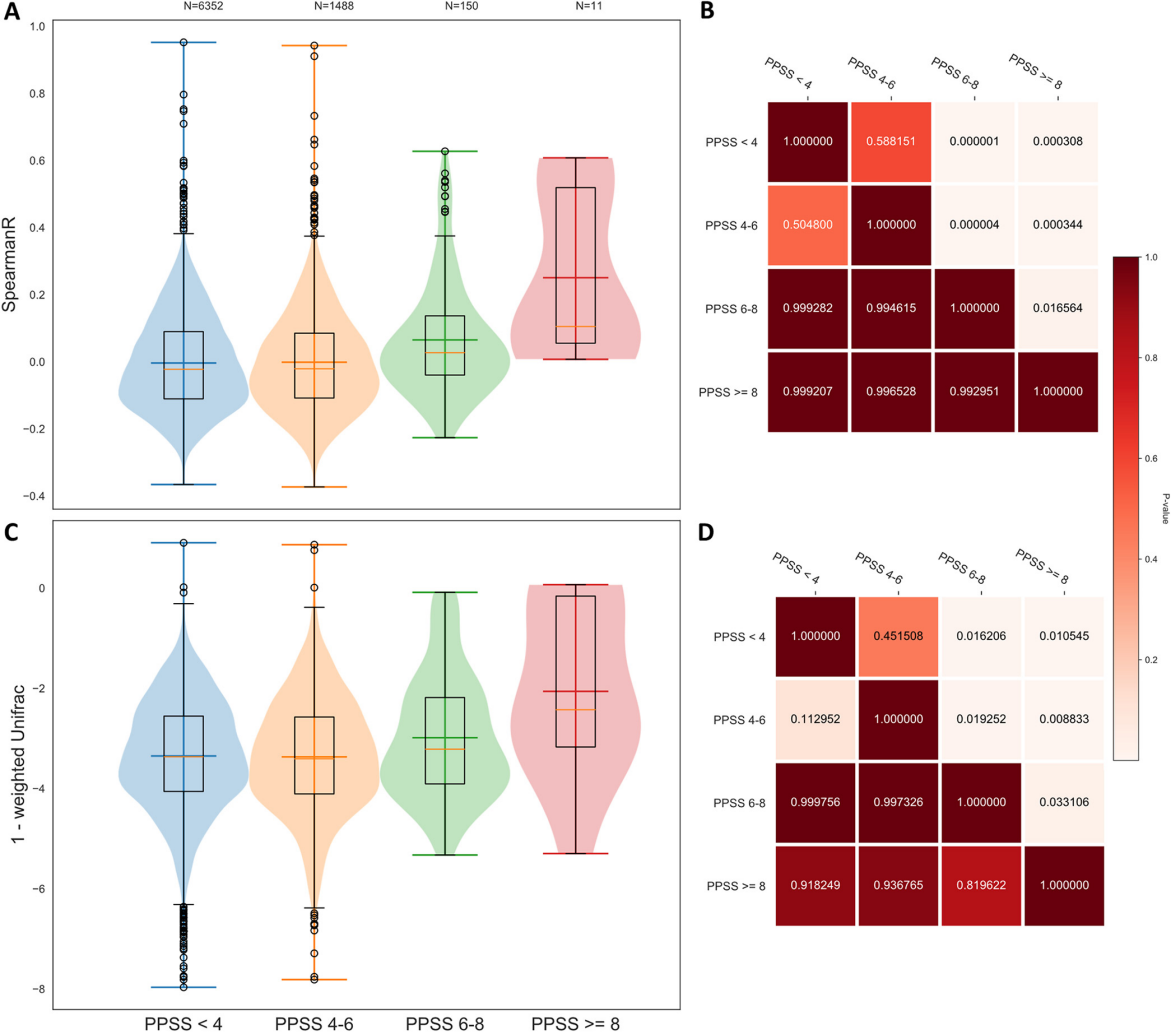
Microbiota beta diversity for sample pairs with increasing PPSS. Spearman rank correlation (A) and weighted UniFrac distance (C) of the microbiota composition for sample pairs with PPSS of <4 (blue), 4 to 6 (orange), 6 to 8 (green), and ≥ 8 (red). (B and D) Heat map displaying the *P* values for the two-sample Kolmogorov-Smirnov test under the null hypothesis that the item in the row is drawn from an equal or smaller distribution compared to the item in the column.

In a second attempt to quantify the functional HLA similarity between individuals, we devised a novel approach specific to human gut microbiota by focusing on the overlap in peptides presented by the different HLA molecules. To do this, we used NetMHCpan4.0, a state-of-the-art peptide-MHC binding affinity prediction tool (44), to predict the binding affinity of over two million gut bacterial oligopeptides to the HLA class I molecules. The peptides were extracted from the human gut catalog (45) in order to best represent the HLA functionality in the context of their potential response to the human gut microbiota. This approach allowed us to calculate a presented peptidome similarity score (PPSS) between the HLA haplotypes of each pair of individuals (see Materials and Methods). Individuals with a high PPSS present very similar peptides, while individuals with a low PPSS present different peptides. When we stratified pairs of individuals by PPSS, we observed a significantly higher microbiota similarity for pairs with a high PPSS (Fig. 2).

### Microbiota alpha diversity is higher in individuals with homozygous HLA alleles.

Although we found that our zygosity predictions were less reliable than our haplotype predictions (Table 1), we still analyzed the effect of zygosity on the microbiota diversity. To this end, we used a subset of data sets of which we could determine the full zygosity of all HLA class I or II genes (*n* = 60 and *n* = 205, respectively). Although we found only very few individuals who were fully homozygous for all HLA genes, we observed a significantly higher microbiota richness in homozygous individuals than in individuals with one or more heterozygous HLA class I genes (Fig. S4A and B). Moreover, we observed a trend of a decrease of the richness as the homozygosity decreased (Fig. S4A and B). Similar results were obtained for HLA class II (Fig. S4E and F). We also investigated the correlation between HLA zygosity and microbiota evenness, but this yielded inconsistent results (Fig. S4C, D, G, and H).

10.1128/mSphere.00476-21.4 FIG S4 Alpha diversity over hetero- and homozygotic HLA class I and II genes. Violin box plots of the Margalef’s richness index for datasets grouped on the number of heterozygote HLA class I (A) and class II (E) genes (71). Violin box plots of the Heip’s evenness measure for individuals stratified on the number of heterozygote HLA class I (C) and II (G) genes (72). (B, D, F, and H) Heat maps display the *P* values for the two-sample Kolmogorov-Smirnov test under the null hypothesis; items on the *y* axis are drawn from an equal or smaller distribution than the item on the *x* axis for HLA class I richness (B), HLA class I evenness (D), HLA class II richness (F) and HLA class II evenness (H). Download FIG S4, TIF file, 1.0 MB. Copyright © 2021 Andeweg et al. 2021 Andeweg et al. https://creativecommons.org/licenses/by/4.0/ This content is distributed under the terms of the Creative Commons Attribution 4.0 International license.

Next, we analyzed whether the zygosity effect on alpha diversity was equally distributed over the HLA class I genes. For HLA class I genes, individuals homozygous for HLA-B genes had a higher richness than HLA-A and HLA-C genes (Fig. S5A). We also found a significant difference for the HLA-DQB1 and HLA-DPB1 class II genes (Fig. S5C). Association between the gut microbiota and HLA-B was observed previously (12, 29) and is consistent with HLA-B often generating immunodominant T cell responses (46–49) and the most polymorphic one (24). We also observed a higher microbiota evenness associated with HLA-DQB1 and -DPB1 homozygosity (Fig. S5D). Finally, we reemphasize that our zygosity predictions might suffer from reduced consistency, and therefore, the above results on the effect of HLA zygosity on human gut microbiota should be interpreted carefully.

10.1128/mSphere.00476-21.5 FIG S5 The influence of homozygosity of different HLA genes on the gut microbiota alpha diversity. Margalef’s richness index (A) and Heip’s evenness measure (B) for individuals who are homozygote for HLA-A (blue), HLA-B (orange), or HLA-C (green) class I genes. HLA-B richness is higher than HLA-A or HLA-C (two-sided two-sample KS test); HLA-A and HLA-C do not differ significantly in richness (two-sided two-sample KS test, *P* = 0.8717). Richness (C) and evenness (D) for individuals who are homozygote for HLA-DQB1 (blue), HLA-DRB1 (orange), or HLA-DPB1 (green) class II genes. Download FIG S5, TIF file, 1.0 MB. Copyright © 2021 Andeweg et al. 2021 Andeweg et al. https://creativecommons.org/licenses/by/4.0/ This content is distributed under the terms of the Creative Commons Attribution 4.0 International license.

## DISCUSSION

We present the first statistical evidence in humans that HLA significantly affects the composition of the gut microbiota, in line with previous results in other animals (18, 20, 28). To do this, we developed the PPSS, a score that uses cutting-edge machine learning tools (44), to quantify the functional similarity between HLA haplotypes of different individuals. We observed the highest correlation between the microbiota and HLA class I profiles using the PPSS, suggesting that this score outperforms simpler measures of HLA type similarity, such as the number of shared alleles between individuals. Thus, if the association holds up in large, well-controlled cohorts, we expect that the PPSS will be useful for comparing HLA/MHC profiles in relation to commensal and pathogenic microorganisms in humans.

HLA class I molecules are involved in intracellular antigen presentation, leading to a cytotoxic response by CD8^+^ T cells. Cross-presentation of extracellular antigens on MHC class I by dendritic cells is common (26, 27, 50, 51), but it remains unknown how antigens presented on HLA class I shape mucosal immunity. In addition, the influence of HLA class I on the microbiota could be related to the MHC class I chain-related antigen A (MIC-A) and antigen B (MIC-B) genes. These polymorphic genes are constitutively expressed in the gut epithelium (52). The recognition of MIC-A/B by its ligand NKG2D, an activating receptor on NK cells, NKT cells, and T cells, leads to cytotoxic response and cytokine production (53). MIC-A and -B are genomically localized next to HLA-B on chromosome 6 and therefore in strong linkage disequilibrium with HLA-B (54, 55). Because we found a microbiota association on HLA class I and the strongest zygosity effect on the gut microbiota for HLA-B (Fig. S5), it will be of interest to dissect the association between HLA-B or MIC-A/B and the microbiota in large cohorts.

MHC class II molecules are suggested to play a role via antibody-mediated response (18, 28, 31). One possibility is that a less diverse HLA repertoire in homozygous individuals may reduce the number and diversity of immune responses to the microbiota by T cells and natural killer (NK) cells. As a result, homozygous individuals would recognize and potentially remove fewer bacterial species from their intestinal microbiota than heterozygous individuals. This could allow a higher number of different bacterial species to colonize the gut than the individuals with a more diverse set of HLA molecules. A similar effect has been observed in the stickleback, where fish with more diverse MHC class II molecules had a less diverse gut microbiota (31). Interestingly, this raises a potential conflict between the fitness benefits of a diverse immune system and of a diverse microbiome. Diverse immune responses are considered beneficial, especially in the protection against rapidly evolving pathogens (56), while a diverse microbiome is considered healthy, since it provides ecological protection against pathogen invasion and avoids dysbiosis (57). Therefore, it remains an open question what the potential effect is of zygosity on the fitness of the host. We focused our analysis on HLA class I molecules because the imputed HLA class II haplotypes did not match the expected population profile and consistency (Table 1 and Fig. S2D and E). Thus, we consider the class II typing less reliable than the class I typing. Moreover, the prediction of presented peptides is not as reliable for class II as the prediction for class I, in part because the binding is more promiscuous for class II than for class I. The seven class II supertypes show a >30% overlap in presented peptides between supertypes, which is similar to the overlap between alleles within a class II supertype (58). We expect that measurements with increased accuracy will reveal similar results with HLA class II haplotypes.

### Conclusion.

An important standing conundrum in microbiome research is the vast interindividual differences in gut microbiome composition between people. While many factors are known that contribute to shaping the microbiome, the relative importance of these factors remains unclear and will be the subject of study for years to come. We expect that innovative data analysis methods, designed to directly probe the functionality of factors such as the human immune system (12, 14), will allow these factors to be elucidated and their contributions mapped. As HLA is a readily measurable parameter of the immune system, the exploration of HLA and HLA-driven peptide presentation opens the door for incorporating these factors into future predictive models of the gut microbiome.

## MATERIALS AND METHODS

### Human gut microbiome data sets.

We retrieved 3,002 human gut data sets from 37 different bioprojects from the public Sequence Read Archive (SRA) database (59). Data sets were collected using “metatranscriptome” search terms (Table 2), but due to inconsistencies in SRA metadata annotation, our selection included 750 metagenomes as well. Microbiota and HLA haplotype profiling are performed on both data types. Repeating the analyses with the subset of 2,250 metatranscriptomes led to a steep decrease in the number of paired individuals with highly similar HLA profiles and loss of signal, and therefore, we decided to continue with the original data set. Metadata fields are listed in Table S2. We are grateful to the associated studies for making these data available for reuse.

**TABLE 2:** Search terms for the SRA query^*a*^

Search term	Filter			No. of data sets
	Source	Organism	Selection	
Human bowel	Metatranscriptomic		Random	841
Human gut	Metatranscriptomic		Random	925
	Transcriptomic	Human gut metagenome orgn txid:408170	Random	130
	Metatranscriptomic	Human gut metagenome orgn txid:408170	Random	897
Human stool	Metatranscriptomic		Random	778
RNA sequencing		Human gut metagenome orgn txid:408170	Random	2,879
Metatranscriptomic		Human gut metagenome orgn txid:408170		2,179

a Overlapping data sets from the different search terms were filtered out.

10.1128/mSphere.00476-21.9 TABLE S2 Additional information on the datasets used. Metadata for the human gut datasets for class I or class II typed datasets. Download Table S2, XLSX file, 0.04 MB. Copyright © 2021 Andeweg et al. 2021 Andeweg et al. https://creativecommons.org/licenses/by/4.0/ This content is distributed under the terms of the Creative Commons Attribution 4.0 International license.

All sequence runs were preprocessed as follows. Data sets were downloaded using prefetch (version 2.9.2) utility, and SRA data were converted to fastq files using fastq-dump (version 2.9.2), both from the SRA toolbox. The quality of the reads was assessed using FastQC (version 0.11.8) (60), and reads were trimmed using AdapterRemoval (version 2.1.7) (61). For studies with multiple data sets from the same individual, we randomly selected one data set to include in our analysis to avoid biasing the data set with intraindividual sample comparisons.

### Mapping reads to a composite reference database.

To assess the microbiota composition and the HLA haplotype at once, reads were mapped with BWA-MEM (version 0.7.12) (62) to a reference database consisting of bacterial small-subunit (SSU) rRNA (SILVA SSU Ref nonredundant [NR] 99, release 132) (63) and genomic HLA sequences from IMGT/HLA database (release 3.31.0, 24). 16S reads were analyzed to obtain the bacterial composition of each data set. Only bacterial operational taxonomic units (OTUs) with a relative abundance of ≥0.0001 were taken into account. Therefore, the data sets should contain ≥1,000 16S reads. We developed a sorting scheme for multimapping reads, which were divided across target sequences as a fraction of the number of primary mapping (unique) reads (64–66).

### HLA imputation.

**FIG 3:**
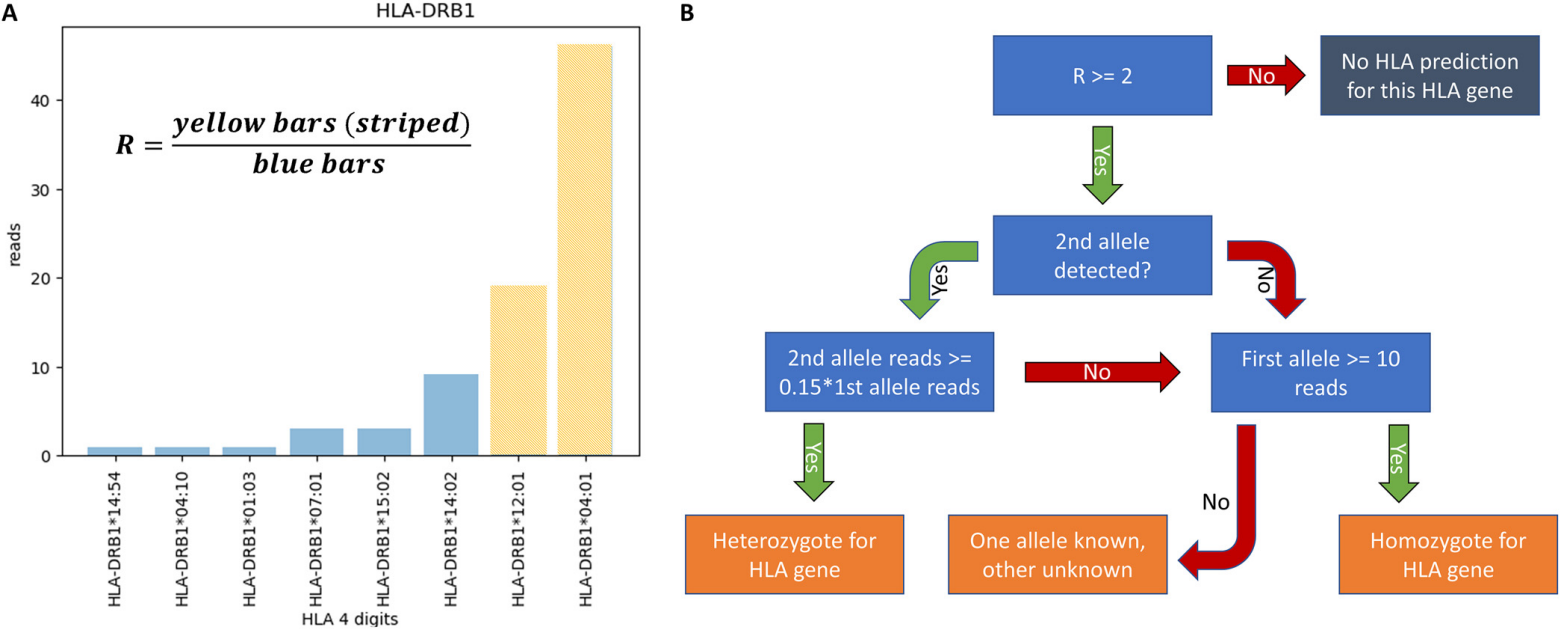
Decision tree genotyping of the HLA gene. (A) Visual explanation of the *R* score. (B) Decision tree for HLA typing from fecal microbiome data sets. Blue boxes show the decisions, orange boxes represent possible positive outcomes, and the gray box shows the negative outcome when no prediction is made for this gene.

We performed two-digit HLA typing on the classical HLA loci (HLA-A, HLA-B, HLA-C, HLA-DR, HLA-DP, and HLA-DQ). Although we expect a maximum of two alleles for each gene (25), we often found more than two HLA alleles with reads mapped. This is likely a result of the high sequence similarity of the different alleles from the same HLA gene. In order to make the most reliable HLA imputation, we defined an *R* score as the ratio of the number of reads mapped to the two most frequently observed HLA alleles over the number of reads mapped to all other alleles of this gene (Fig. 3A). We determined *R* for each of the six classical HLA loci in all 3,002 data sets. Only HLA genes with an *R* score of 2 or higher were considered for HLA imputation (Fig. 3B). An individual was considered homozygotic at a given locus if at least 10 reads aligned to the top HLA allele and the second mapped allele had less than 15% of the reads of the first. A locus was considered heterozygotic if at least two alleles were found and the second mapped to at least 15% of the reads of the first. This number was optimized based on the highest number of correct predictions in data sets originating from the same individual (Table S1).

10.1128/mSphere.00476-21.8 TABLE S1 HLA dependence of consistency of HLA prediction on zygosity cutoff. Number of correct allele predictions for different zygosity determining cutoffs. Download Table S1, XLSX file, 0.01 MB. Copyright © 2021 Andeweg et al. 2021 Andeweg et al. https://creativecommons.org/licenses/by/4.0/ This content is distributed under the terms of the Creative Commons Attribution 4.0 International license.

### Data set selection and population-based HLA typing quality control.

Individual HLA loci may be in linkage disequilibrium with other loci (67, 68). To maximize the representation of the HLA profile, we included data sets only if the complete HLA haplotype was assessed, resulting in two groups for the classical class I and II (*n* = 127 and 309, respectively) (Table S2).

Quality control of our haplotyping approach was performed by comparing the population allele and zygosity frequencies of our data with the NMDP High-Resolution HLA Alleles and Haplotypes in the U.S. Population database (41; https://bioinformatics.bethematchclinical.org/hla-resources/haplotype-frequencies/high-resolution-hla-alleles-and-haplotypes-in-the-us-population/). Data were extracted in September 2019. Based on the data set localities, we expected a majority of individuals in our data sets to be of European descent (Table S2). Therefore, we compared our HLA frequencies with the cohort of European origin in NMDP database. A second quality control approach was performed using multiple data sets originating from the same individual; this information was obtained from the metadata. In the final analysis, only one data set per individual was used.

### HLA supertypes and PPSS.

Most of the 450 haplotyped data sets had unique HLA profiles, reflective of the high population-level diversity at the HLA loci. To increase statistical sensitivity, we performed two separate experiments on the data. First, we clustered HLA-A and HLA-B alleles into six supertypes each (42). Second, we devised a presented peptidome similarity score (PPSS) between two haplotypes to estimate the functional similarity in immune profile, based on the overlap in peptides presented by their HLAs. To do this, first we created a peptidome data set consisting of human gut bacterial peptides sampled from the 9,878,647 proteins in the human gut catalogue (45). By using a sliding window of length nine amino acids, we randomly included one in every thousand oligopeptides into the peptidome and removed duplicates, resulting in a peptidome of 2,371,920 peptides. Next, we used NetMHCpan4.0 to predict which peptides were presented by each HLA class I allele (44). We used the recommended cutoff 2.0%Rank for classifying binding peptides.

The PPSS between two individuals, *i* and *j*, is defined as the summed Jaccard indices, *J_g,ai,aj_*, of the sets of peptides presented by all combinations of the alleles *ai* and *aj* of HLA gene *g* (HLA-A, HLA-B, and HLA-C). The PPSS ranges from 0 (no overlap between presented peptides) to 12 (presented peptides are identical). The Jaccard-based similarity of peptide pools presented of allele pair *a*,*b* is defined as the overlap of peptide pools (*O_a_*_,_*_b_*) divided by the not-shared peptide pools (equation 1).

(1) $$J_{\mathrm{ab}}=\frac{O_{a,b}}{\left(P_{a}+P_{b}-O_{a,b}\right)}$$

The *J_ab_* scores between all two-digit alleles are shown in Fig. S6. PPSS*_ij_*, the similarity of peptide pools of the pair of individuals *i* and *j*, is defined by the allele pair similarity for all combinations of alleles within one gene, added up for all three HLA genes (equation 2).

(2) $${\text{PPSS}}_{\mathrm{ij}}=\underset{g=A,\;B,\;C}{\sum }\underset{\mathrm{ai}=1,\;2}{\sum }\underset{\mathrm{aj}=1,\;2}{\sum }J_{g,\mathrm{ai},\mathrm{aj}}$$

The distribution of PPSS scores across all data sets is shown in Fig. S7.

10.1128/mSphere.00476-21.6 FIG S6 Jaccard peptide presentation score between different HLA molecules for HLA-A (A), HLA-B (B), and HLA-C (C) genes. Download FIG S6, TIF file, 1.5 MB. Copyright © 2021 Andeweg et al. 2021 Andeweg et al. https://creativecommons.org/licenses/by/4.0/ This content is distributed under the terms of the Creative Commons Attribution 4.0 International license.

10.1128/mSphere.00476-21.7 FIG S7 Histogram of PPSS pairs of individuals. The bar colors represent the stratification used in Fig. 2; sample pairs with a PPSS of <4 (blue), 4 to 6 (orange), 6 to 8 (green), and ≥8 (red). Download FIG S7, TIF file, 0.8 MB. Copyright © 2021 Andeweg et al. 2021 Andeweg et al. https://creativecommons.org/licenses/by/4.0/ This content is distributed under the terms of the Creative Commons Attribution 4.0 International license.

Data Set S1 contains the Jaccard indices for every pair of four-digit alleles for HLA gene HLA-A, HLA-B, and HLA-C, allowing readers to readily calculate the PPSS upon HLA typing.

10.1128/mSphere.00476-21.10 DATA SET S1 PPSS for all possible four-digit allele pairs within each class I HLA gene. Download Data Set S1, XLSX file, 18.2 MB. Copyright © 2021 Andeweg et al. 2021 Andeweg et al. https://creativecommons.org/licenses/by/4.0/ This content is distributed under the terms of the Creative Commons Attribution 4.0 International license.

### Alpha diversity.

To assess alpha diversity, we used evenness and richness measures from the skbio.diversity.alpha package (version 0.5.5) (69). We did not rarefy the data, as this may increase type I and II errors (70). We did not find a correlation between data set size and microbiota richness (*r* = 0.027; *P* = 0.16, Pearson), and we therefore assume that data set size does not bias our results. To approximate the number of different species in a sample, we used Margalef’s richness index (*D*_mg_), which attenuates sampling size effects (equation 3) (71), where *S* is the number of different OTUs observed in the sample and *N* is the number of 16S reads mapped:

(3) $$D_{\text{mg}}=\frac{\left(S-1\right)}{\text{ln}\;N}$$

The microbiota evenness was calculated using Heip’s evenness measure (*E*) (equation 4) (72), where *H* is the Shannon-Weiner entropy of the 16S reads and *S* is the number of OTUs in the data set:

(4) $$E=\frac{\left(e^{H}-1\right)}{\left(S-1\right)}$$

For the comparison between the alpha diversity and the number of heterozygote genes, only the fully classified data sets were used (Table S2). In the analysis of the effect of homozygous HLA genes on alpha diversity, all data sets with at least one homozygous gene were used.

### Beta diversity.

The microbiota similarity between individuals was calculated using two beta diversity measures: weighted UniFrac distance and Spearman’s rank correlation (73). We adapted the SILVA taxonomy with a branch length of 1 between taxonomic ranks and used these distances for weighted UniFrac. The Spearman rank correlation was calculated using the abundances of the OTUs observed in each microbiome.

### Statistics.

We used the Kolmogorov-Smirnov (KS) test for two samples from the python package scipy.stats (version 1.3.2) (74) for statistical analysis, with alternative hypothesis, “two-sided,” “less,” and “greater,” depending on the relevance for the comparison, as stated in the figure legends.

### Code availability.

Scripts for downloading the data sets, mapping the data sets to the composed reference genome, and further analysis can be accessed on GitHub (github.com/Stijn-A/HLA_microbiome).
