# Supplementary figures and images for: Quantifying the Impact of Human Leukocyte Antigen on the Human Gut Microbiota
Source: mSphere. 2021 Aug 11;6(4):e00476-21. doi: 10.1128/mSphere.00476-21 (PMC8386457; doi:10.1128/mSphere.00476-21)

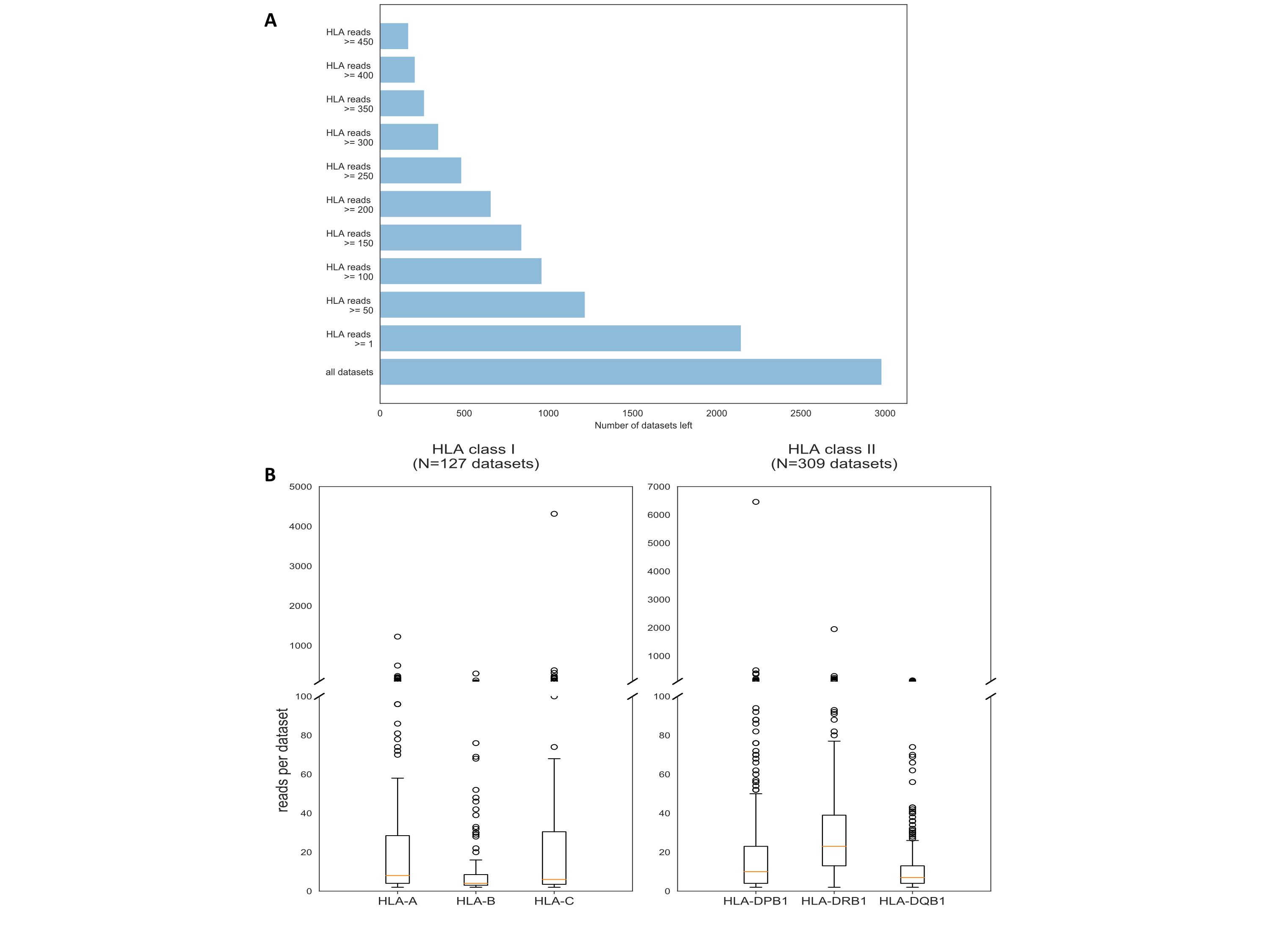

Supplement: FIG S1 [file msphere.00476-21-sf001.tif]

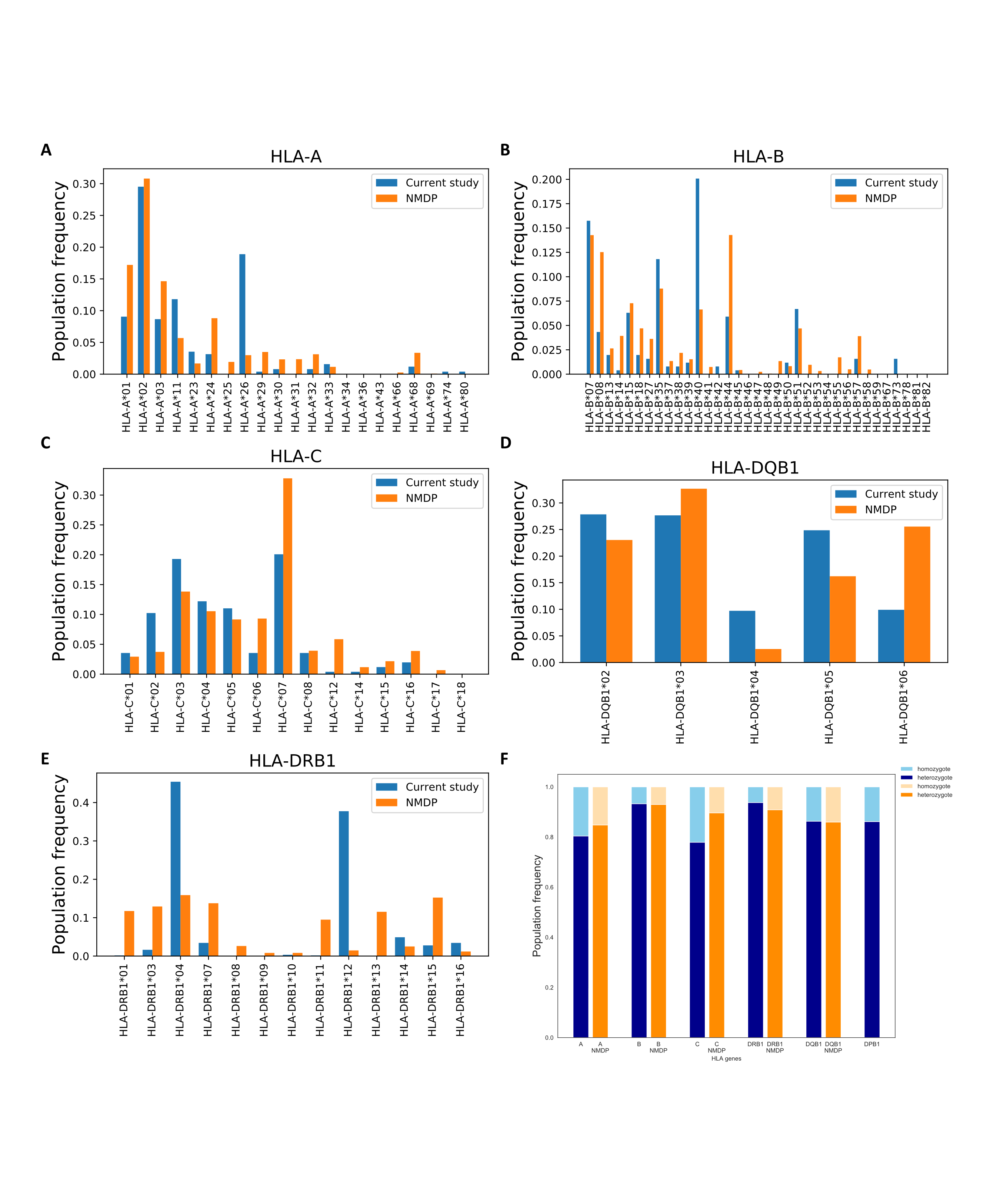

Supplement: FIG S2 [file msphere.00476-21-sf002.tif]

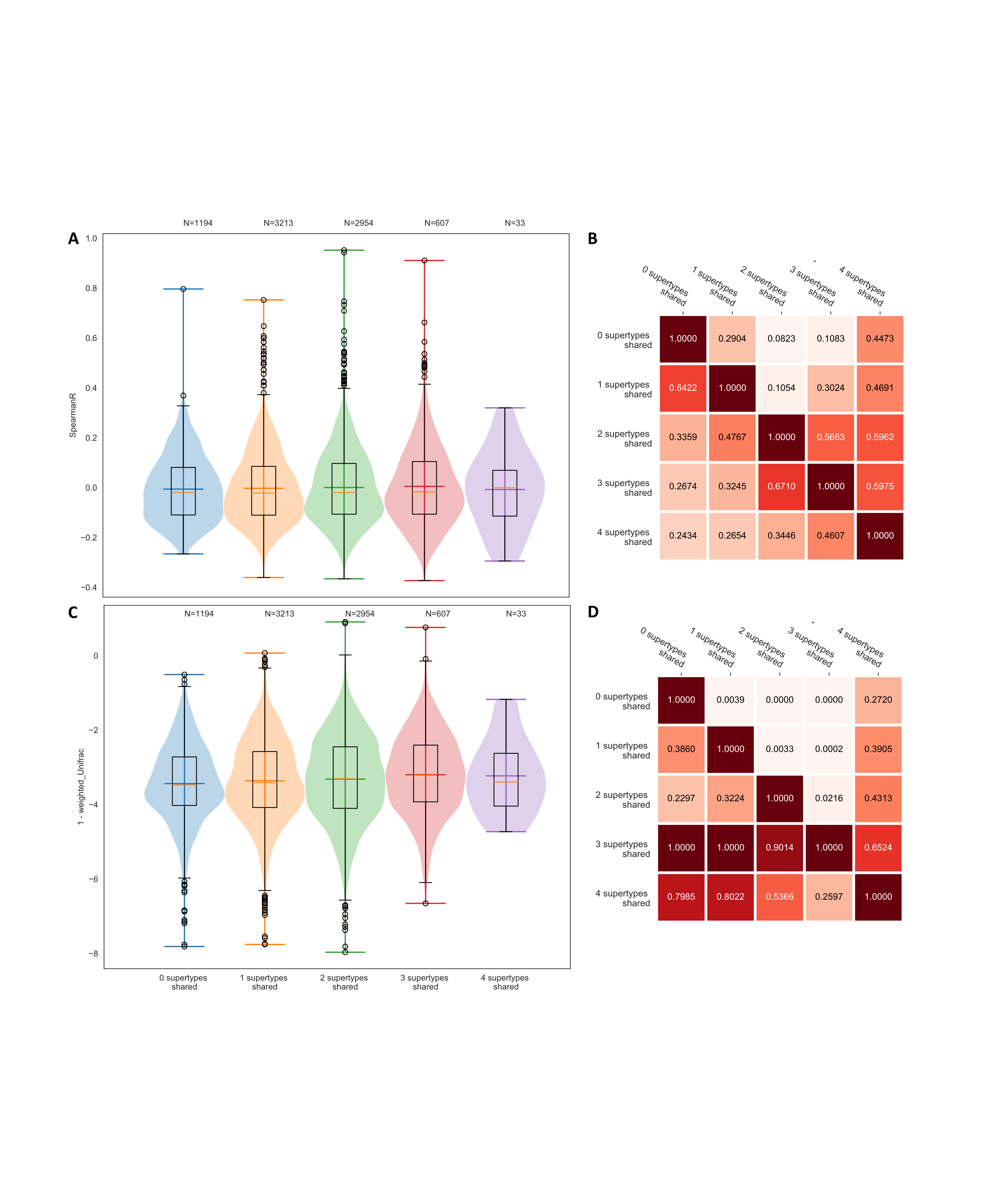

Supplement: FIG S3 [file msphere.00476-21-sf003.tif]

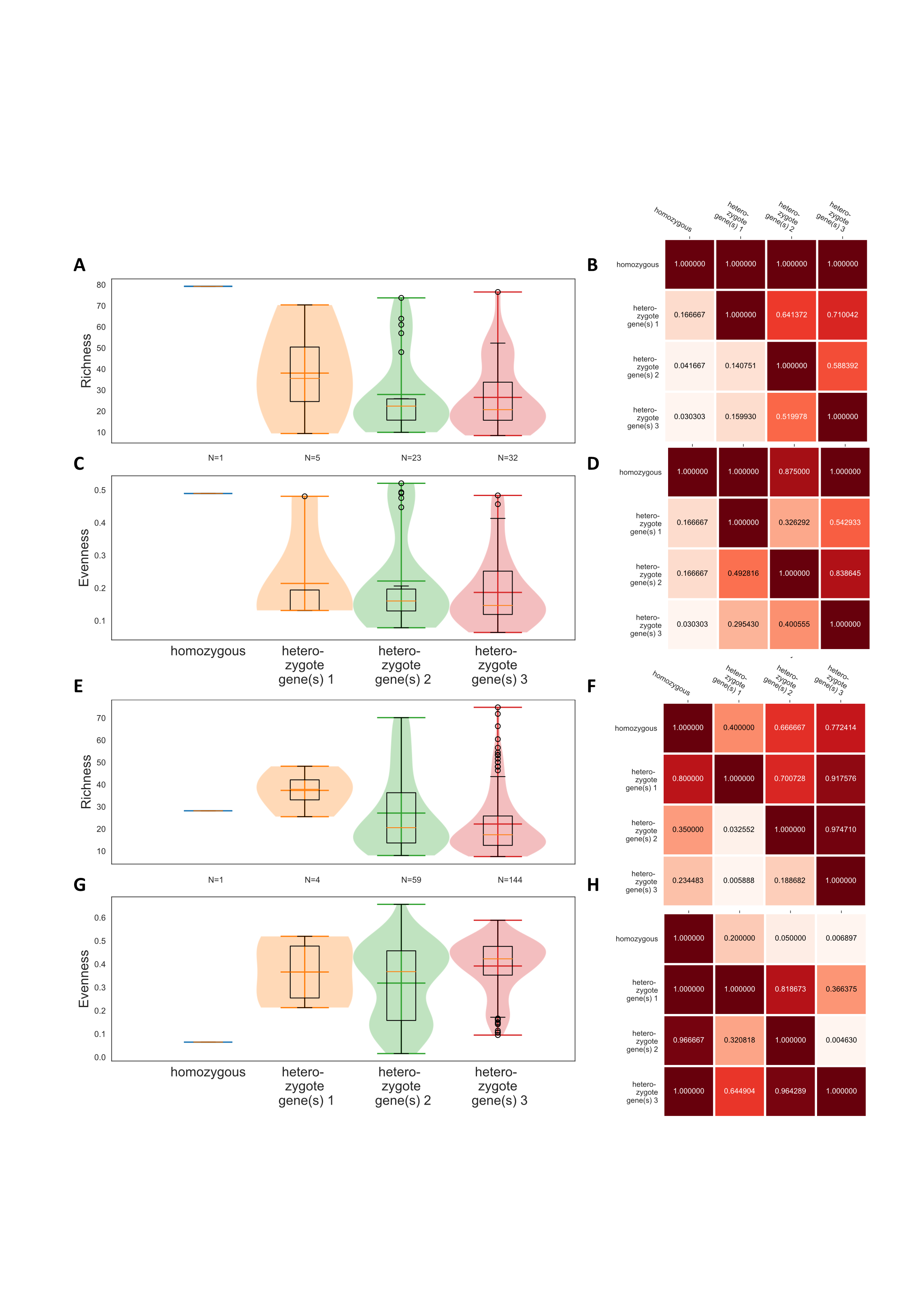

Supplement: FIG S4 [file msphere.00476-21-sf004.tif]

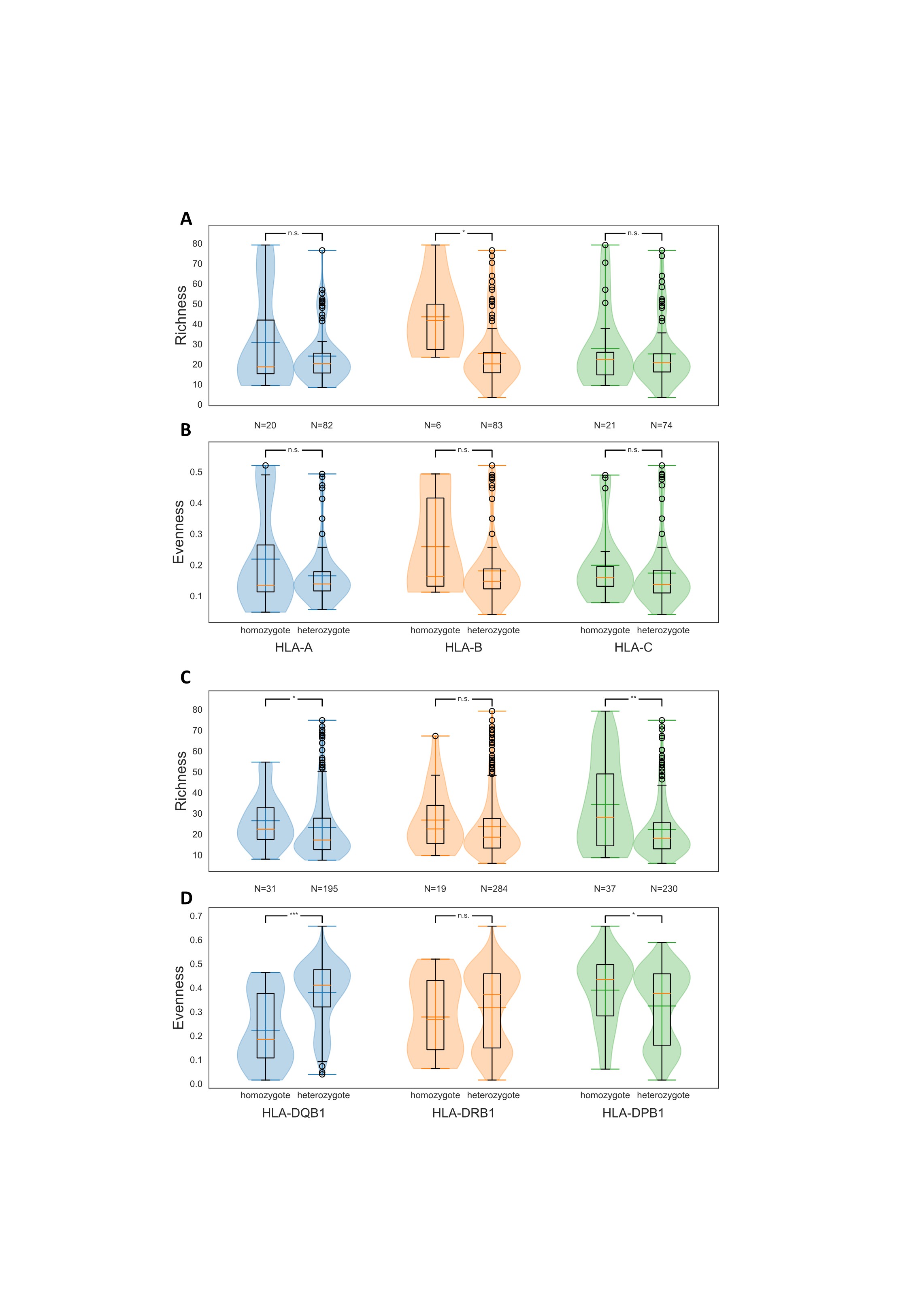

Supplement: FIG S5 [file msphere.00476-21-sf005.tif]

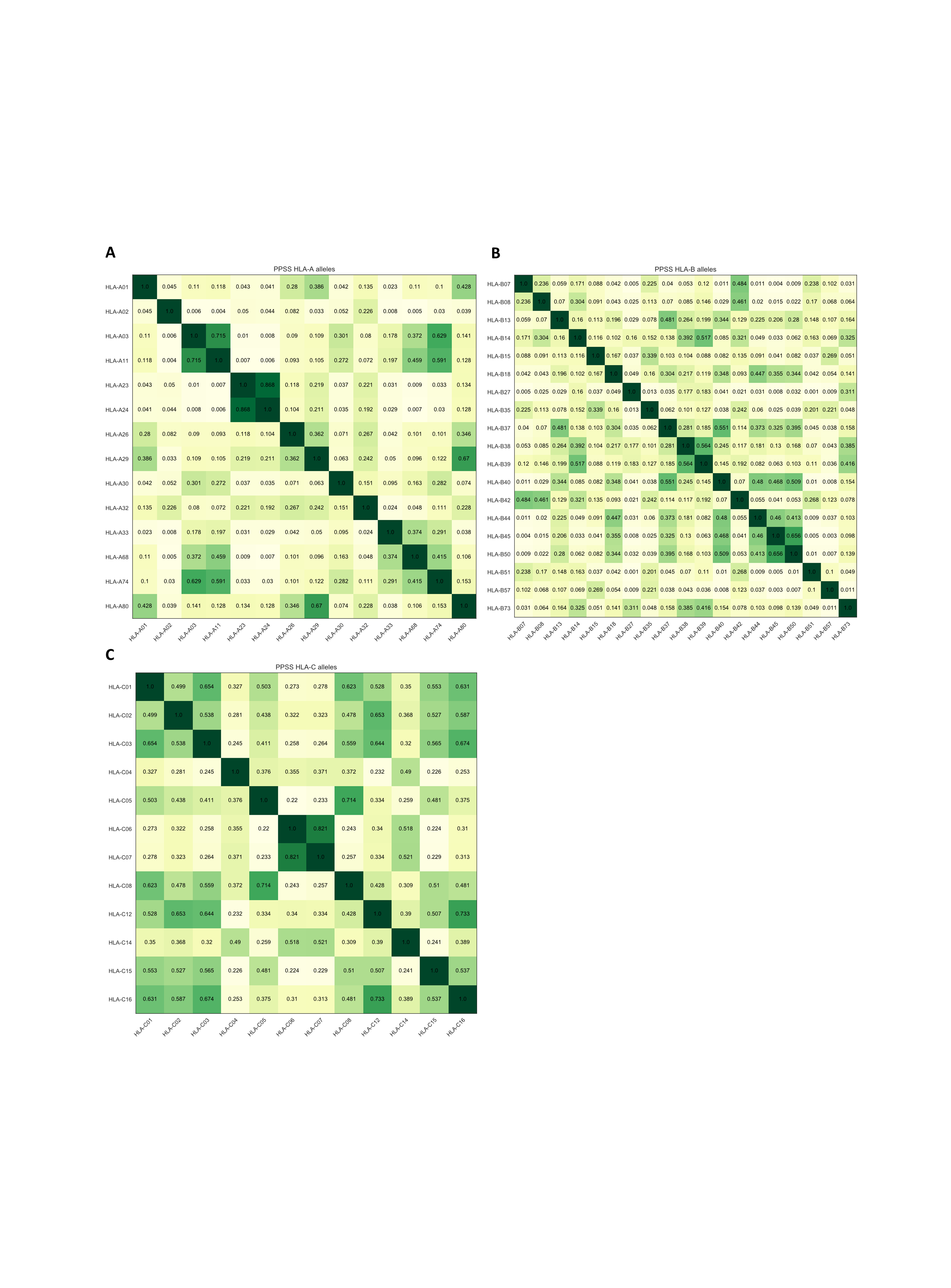

Supplement: FIG S6 [file msphere.00476-21-sf006.tif]

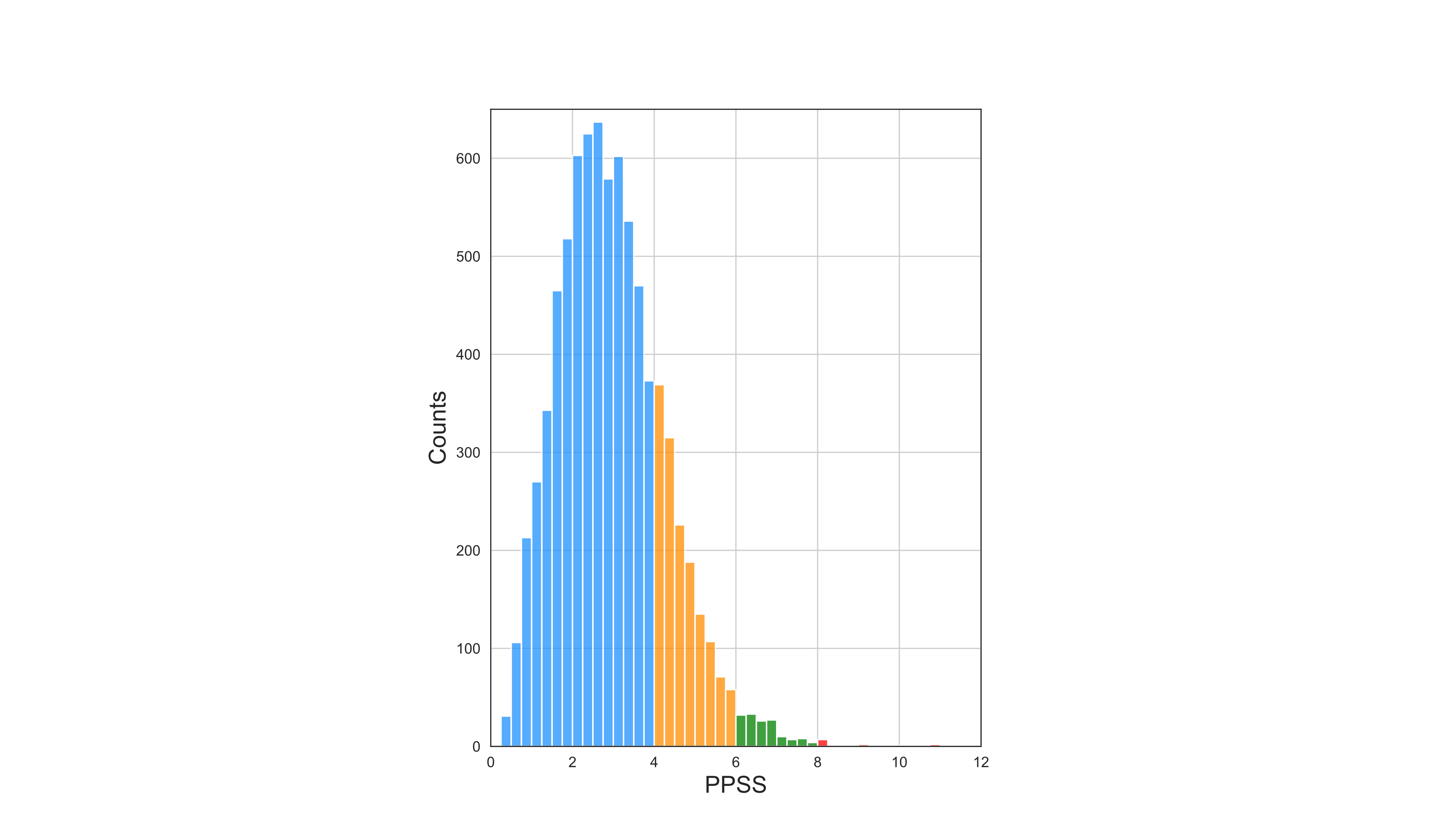

Supplement: FIG S7 [file msphere.00476-21-sf007.tif]
